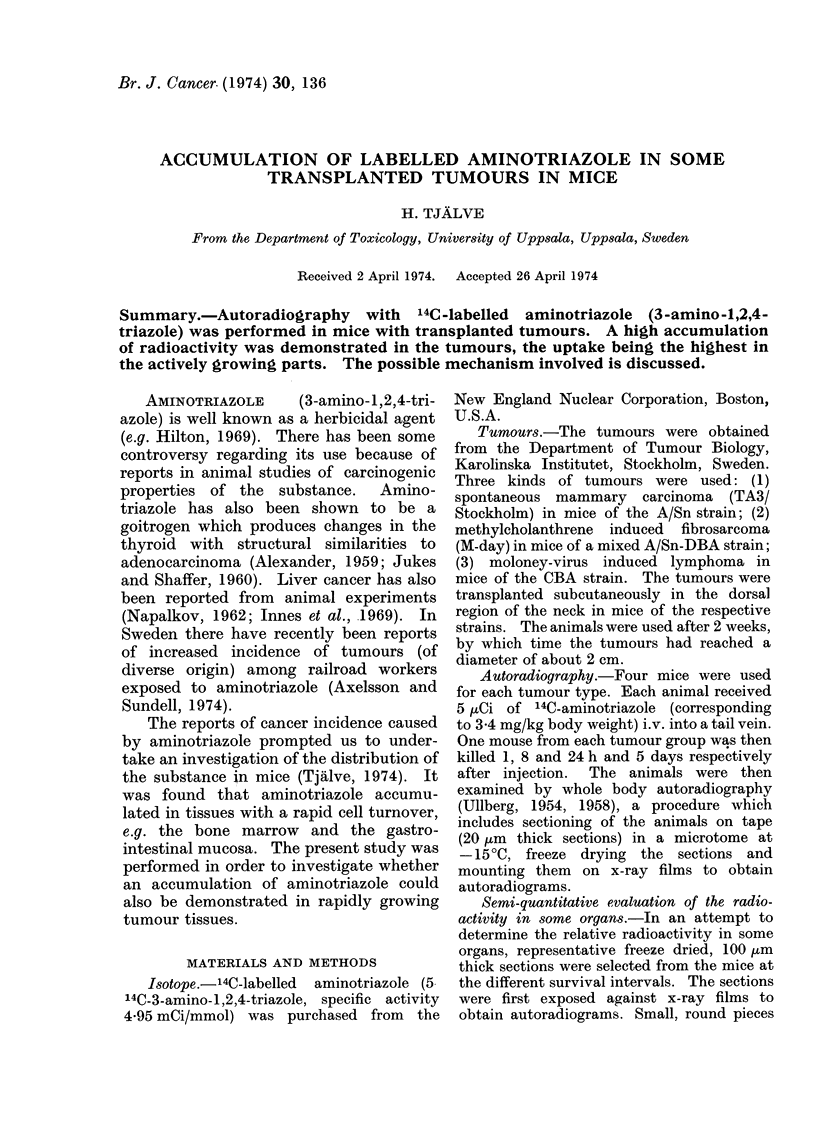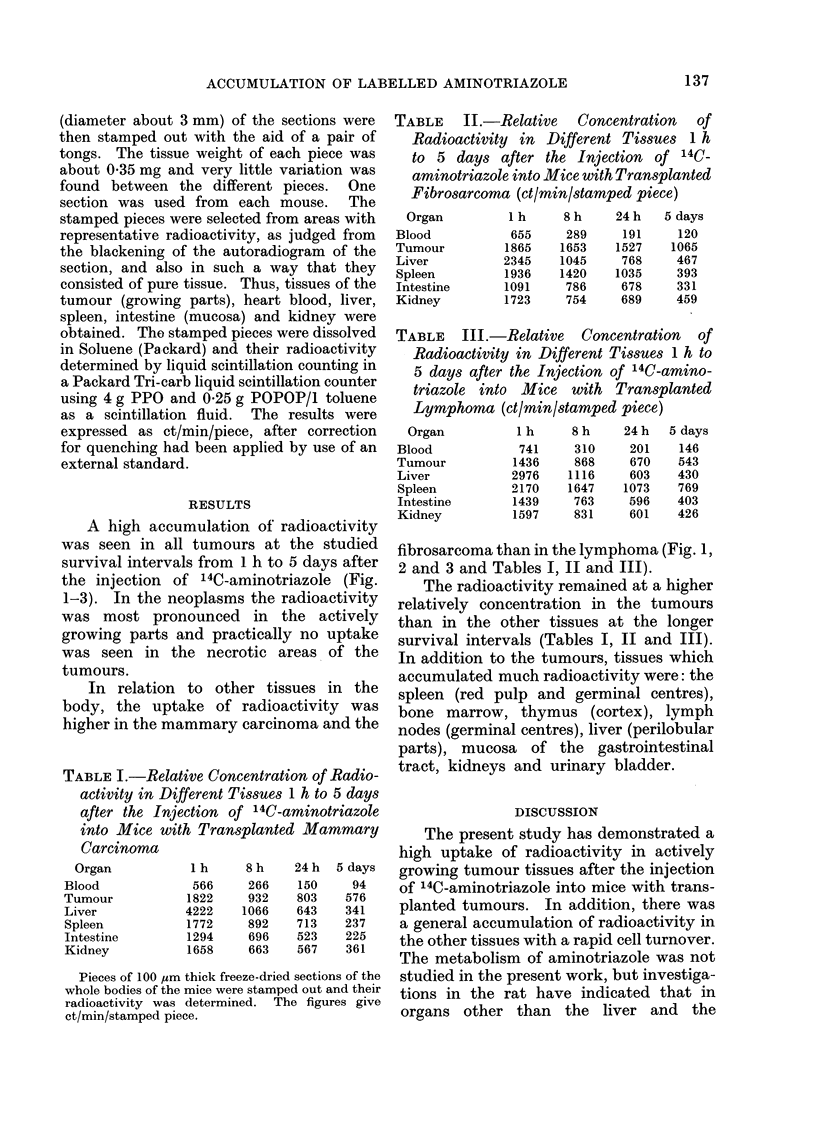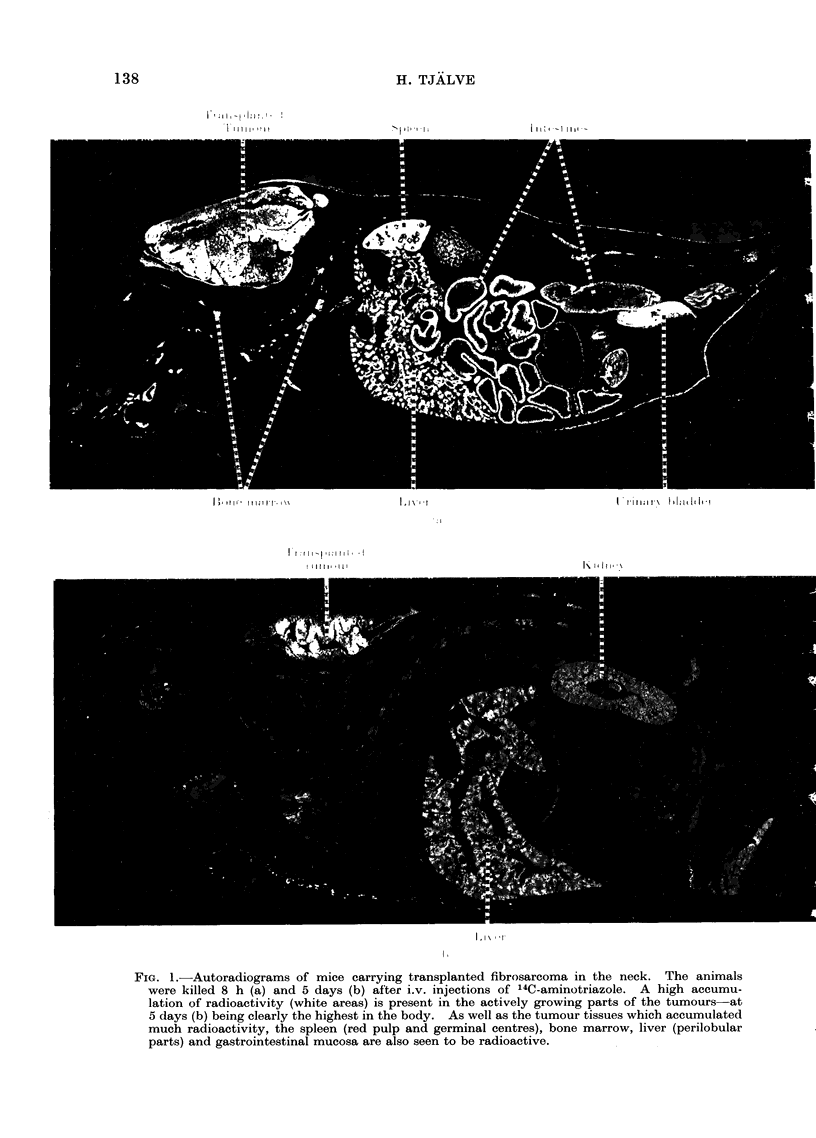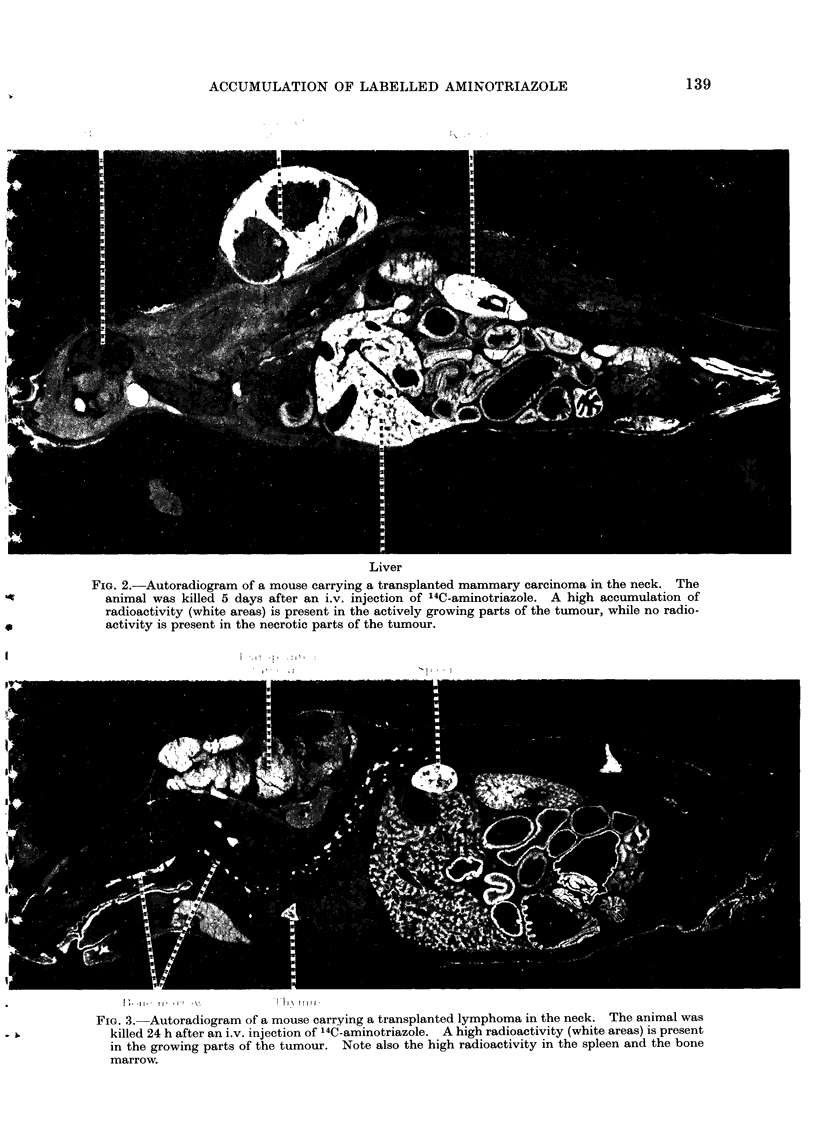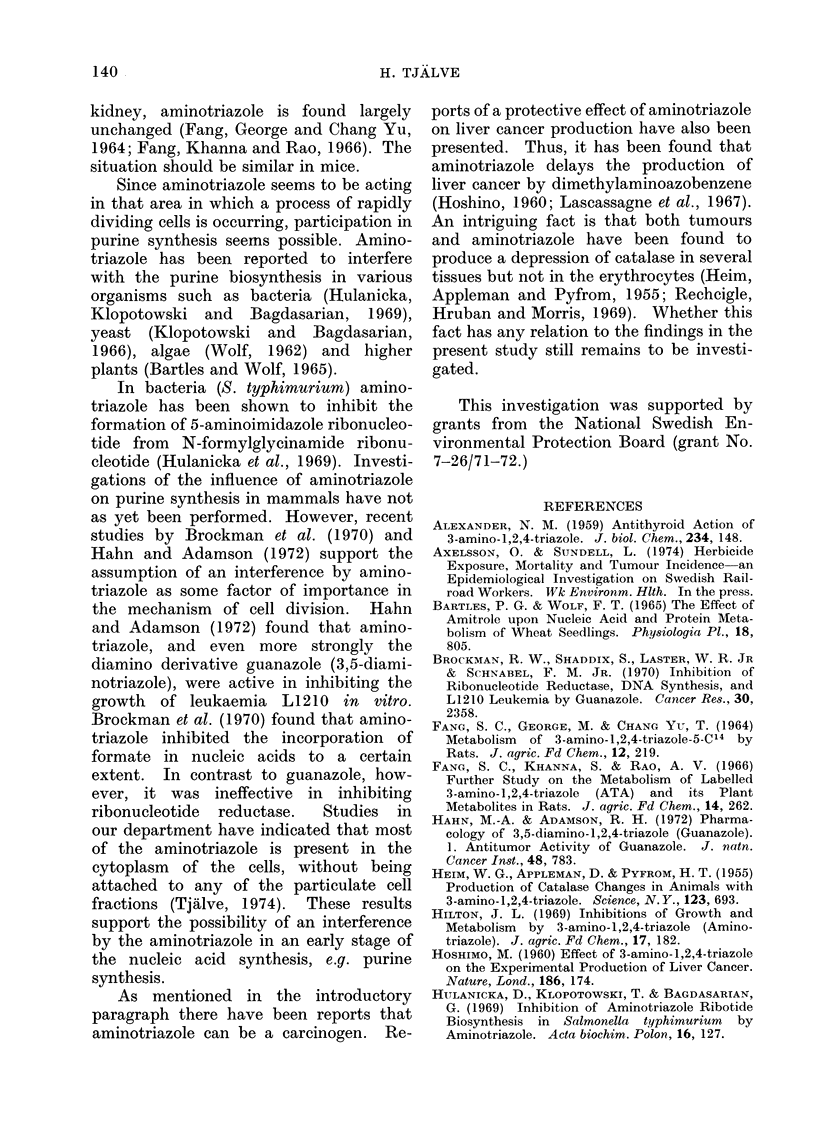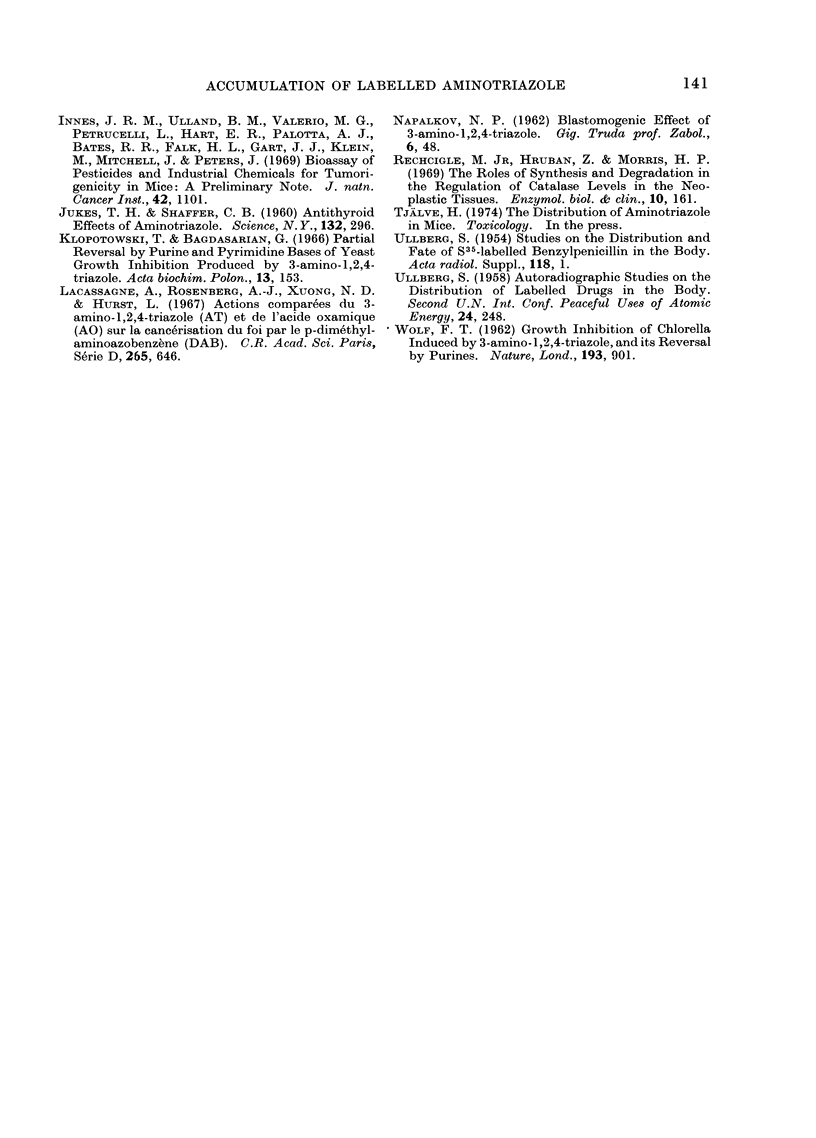# Accumulation of Labelled Aminotriazole in Some Transplanted Tumours in Mice

**DOI:** 10.1038/bjc.1974.124

**Published:** 1974-08

**Authors:** H. Tjälve

## Abstract

**Images:**


*Br. J. Cancer* (1974) **30**, 136

### ACCUMULATION OF LABELLED AMINOTRIAZOLE IN SOME TRANSPLANTED TUMOURS IN MICE

H. TJÄLVE

*From the Department of Toxicology, University of Uppsala, Uppsala, Sweden*

Received 2 April 1974. Accepted 26 April 1974

**Summary.—Autoradiography with $^{14}C$-labelled aminotriazole (3-amino-1,2,4-triazole) was performed in mice with transplanted tumours. A high accumulation of radioactivity was demonstrated in the tumours, the uptake being the highest in the actively growing parts. The possible mechanism involved is discussed.**

AMINOTRIAZOLE (3-amino-1,2,4-triazole) is well known as a herbicidal agent (*e.g.* Hilton, 1969). There has been some controversy regarding its use because of reports in animal studies of carcinogenic properties of the substance. Aminotriazole has also been shown to be a goitrogen which produces changes in the thyroid with structural similarities to adenocarcinoma (Alexander, 1959; Jukes and Shaffer, 1960). Liver cancer has also been reported from animal experiments (Napalkov, 1962; Innes *et al.*, 1969). In Sweden there have recently been reports of increased incidence of tumours (of diverse origin) among railroad workers exposed to aminotriazole (Axelsson and Sundell, 1974).

The reports of cancer incidence caused by aminotriazole prompted us to undertake an investigation of the distribution of the substance in mice (Tjälve, 1974). It was found that aminotriazole accumulated in tissues with a rapid cell turnover, *e.g.* the bone marrow and the gastrointestinal mucosa. The present study was performed in order to investigate whether an accumulation of aminotriazole could also be demonstrated in rapidly growing tumour tissues.

### MATERIALS AND METHODS

*Isotope.*—$^{14}C$-labelled aminotriazole (5-$^{14}C$-3-amino-1,2,4-triazole, specific activity 4·95 mCi/mmol) was purchased from the New England Nuclear Corporation, Boston, U.S.A.

*Tumours.*—The tumours were obtained from the Department of Tumour Biology, Karolinska Institutet, Stockholm, Sweden. Three kinds of tumours were used: (1) spontaneous mammary carcinoma (TA3/Stockholm) in mice of the A/Sn strain; (2) methylcholanthrene induced fibrosarcoma (M-day) in mice of a mixed A/Sn-DBA strain; (3) moloney-virus induced lymphoma in mice of the CBA strain. The tumours were transplanted subcutaneously in the dorsal region of the neck in mice of the respective strains. The animals were used after 2 weeks, by which time the tumours had reached a diameter of about 2 cm.

*Autoradiography.*—Four mice were used for each tumour type. Each animal received 5 $\mu$Ci of $^{14}C$-aminotriazole (corresponding to 3·4 mg/kg body weight) i.v. into a tail vein. One mouse from each tumour group was then killed 1, 8 and 24 h and 5 days respectively after injection. The animals were then examined by whole body autoradiography (Ullberg, 1954, 1958), a procedure which includes sectioning of the animals on tape (20 $\mu$m thick sections) in a microtome at $-15$°C, freeze drying the sections and mounting them on x-ray films to obtain autoradiograms.

*Semi-quantitative evaluation of the radioactivity in some organs.*—In an attempt to determine the relative radioactivity in some organs, representative freeze dried, 100 $\mu$m thick sections were selected from the mice at the different survival intervals. The sections were first exposed against x-ray films to obtain autoradiograms. Small, round pieces

(diameter about 3 mm) of the sections were then stamped out with the aid of a pair of tongs. The tissue weight of each piece was about 0·35 mg and very little variation was found between the different pieces. One section was used from each mouse. The stamped pieces were selected from areas with representative radioactivity, as judged from the blackening of the autoradiogram of the section, and also in such a way that they consisted of pure tissue. Thus, tissues of the tumour (growing parts), heart blood, liver, spleen, intestine (mucosa) and kidney were obtained. The stamped pieces were dissolved in Soluene (Packard) and their radioactivity determined by liquid scintillation counting in a Packard Tri-carb liquid scintillation counter using 4 g PPO and 0·25 g POPOP/l toluene as a scintillation fluid. The results were expressed as ct/min/piece, after correction for quenching had been applied by use of an external standard.

### RESULTS

A high accumulation of radioactivity was seen in all tumours at the studied survival intervals from 1 h to 5 days after the injection of $^{14}C$-aminotriazole (Fig. 1–3). In the neoplasms the radioactivity was most pronounced in the actively growing parts and practically no uptake was seen in the necrotic areas of the tumours.

In relation to other tissues in the body, the uptake of radioactivity was higher in the mammary carcinoma and the fibrosarcoma than in the lymphoma (Fig. 1, 2 and 3 and Tables I, II and III).

The radioactivity remained at a higher relatively concentration in the tumours than in the other tissues at the longer survival intervals (Tables I, II and III). In addition to the tumours, tissues which accumulated much radioactivity were: the spleen (red pulp and germinal centres), bone marrow, thymus (cortex), lymph nodes (germinal centres), liver (perilobular parts), mucosa of the gastrointestinal tract, kidneys and urinary bladder.

TABLE I.—*Relative Concentration of Radioactivity in Different Tissues 1 h to 5 days after the Injection of $^{14}C$-aminotriazole into Mice with Transplanted Mammary Carcinoma*

| Organ | 1 h | 8 h | 24 h | 5 days |
|---|---|---|---|---|
| Blood | 566 | 266 | 150 | 94 |
| Tumour | 1822 | 932 | 803 | 576 |
| Liver | 4222 | 1066 | 643 | 341 |
| Spleen | 1772 | 892 | 713 | 237 |
| Intestine | 1294 | 696 | 523 | 225 |
| Kidney | 1658 | 663 | 567 | 361 |

Pieces of 100 μm thick freeze-dried sections of the whole bodies of the mice were stamped out and their radioactivity was determined. The figures give ct/min/stamped piece.

TABLE II.—*Relative Concentration of Radioactivity in Different Tissues 1 h to 5 days after the Injection of $^{14}C$-aminotriazole into Mice with Transplanted Fibrosarcoma (ct/min/stamped piece)*

| Organ | 1 h | 8 h | 24 h | 5 days |
|---|---|---|---|---|
| Blood | 655 | 289 | 191 | 120 |
| Tumour | 1865 | 1653 | 1527 | 1065 |
| Liver | 2345 | 1045 | 768 | 467 |
| Spleen | 1936 | 1420 | 1035 | 393 |
| Intestine | 1091 | 786 | 678 | 331 |
| Kidney | 1723 | 754 | 689 | 459 |

TABLE III.—*Relative Concentration of Radioactivity in Different Tissues 1 h to 5 days after the Injection of $^{14}C$-aminotriazole into Mice with Transplanted Lymphoma (ct/min/stamped piece)*

| Organ | 1 h | 8 h | 24 h | 5 days |
|---|---|---|---|---|
| Blood | 741 | 310 | 201 | 146 |
| Tumour | 1436 | 868 | 670 | 543 |
| Liver | 2976 | 1116 | 603 | 430 |
| Spleen | 2170 | 1647 | 1073 | 769 |
| Intestine | 1439 | 763 | 596 | 403 |
| Kidney | 1597 | 831 | 601 | 426 |

### DISCUSSION

The present study has demonstrated a high uptake of radioactivity in actively growing tumour tissues after the injection of $^{14}C$-aminotriazole into mice with transplanted tumours. In addition, there was a general accumulation of radioactivity in the other tissues with a rapid cell turnover. The metabolism of aminotriazole was not studied in the present work, but investigations in the rat have indicated that in organs other than the liver and the

FIG. 1.—Autoradiograms of mice carrying transplanted fibrosarcoma in the neck. The animals were killed 8 h (a) and 5 days (b) after i.v. injections of $^{14}$C-aminotriazole. A high accumulation of radioactivity (white areas) is present in the actively growing parts of the tumours—at 5 days (b) being clearly the highest in the body. As well as the tumour tissues which accumulated much radioactivity, the spleen (red pulp and germinal centres), bone marrow, liver (perilobular parts) and gastrointestinal mucosa are also seen to be radioactive.

FIG. 2.—Autoradiogram of a mouse carrying a transplanted mammary carcinoma in the neck. The animal was killed 5 days after an i.v. injection of $^{14}C$-aminotriazole. A high accumulation of radioactivity (white areas) is present in the actively growing parts of the tumour, while no radioactivity is present in the necrotic parts of the tumour.

FIG. 3.—Autoradiogram of a mouse carrying a transplanted lymphoma in the neck. The animal was killed 24 h after an i.v. injection of $^{14}C$-aminotriazole. A high radioactivity (white areas) is present in the growing parts of the tumour. Note also the high radioactivity in the spleen and the bone marrow.

kidney, aminotriazole is found largely unchanged (Fang, George and Chang Yu, 1964; Fang, Khanna and Rao, 1966). The situation should be similar in mice.

Since aminotriazole seems to be acting in that area in which a process of rapidly dividing cells is occurring, participation in purine synthesis seems possible. Aminotriazole has been reported to interfere with the purine biosynthesis in various organisms such as bacteria (Hulanicka, Klopotowski and Bagdasarian, 1969), yeast (Klopotowski and Bagdasarian, 1966), algae (Wolf, 1962) and higher plants (Bartles and Wolf, 1965).

In bacteria (*S. typhimurium*) aminotriazole has been shown to inhibit the formation of 5-aminoimidazole ribonucleotide from N-formylglycinamide ribonucleotide (Hulanicka *et al.*, 1969). Investigations of the influence of aminotriazole on purine synthesis in mammals have not as yet been performed. However, recent studies by Brockman *et al.* (1970) and Hahn and Adamson (1972) support the assumption of an interference by aminotriazole as some factor of importance in the mechanism of cell division. Hahn and Adamson (1972) found that aminotriazole, and even more strongly the diamino derivative guanazole (3,5-diaminotriazole), were active in inhibiting the growth of leukaemia L1210 *in vitro*. Brockman *et al.* (1970) found that aminotriazole inhibited the incorporation of formate in nucleic acids to a certain extent. In contrast to guanazole, however, it was ineffective in inhibiting ribonucleotide reductase. Studies in our department have indicated that most of the aminotriazole is present in the cytoplasm of the cells, without being attached to any of the particulate cell fractions (Tjälve, 1974). These results support the possibility of an interference by the aminotriazole in an early stage of the nucleic acid synthesis, *e.g.* purine synthesis.

As mentioned in the introductory paragraph there have been reports that aminotriazole can be a carcinogen. Reports of a protective effect of aminotriazole on liver cancer production have also been presented. Thus, it has been found that aminotriazole delays the production of liver cancer by dimethylaminoazobenzene (Hoshino, 1960; Lascassagne *et al.*, 1967). An intriguing fact is that both tumours and aminotriazole have been found to produce a depression of catalase in several tissues but not in the erythrocytes (Heim, Appleman and Pyfrom, 1955; Rechcigle, Hruban and Morris, 1969). Whether this fact has any relation to the findings in the present study still remains to be investigated.

This investigation was supported by grants from the National Swedish Environmental Protection Board (grant No. 7–26/71–72.)

### REFERENCES

ALEXANDER, N. M. (1959) Antithyroid Action of 3-amino-1,2,4-triazole. *J. biol. Chem.*, **234**, 148.

AXELSSON, O. & SUNDELL, L. (1974) Herbicide Exposure, Mortality and Tumour Incidence—an Epidemiological Investigation on Swedish Railroad Workers. *Wk Environm. Hlth.* In the press.

BARTLES, P. G. & WOLF, F. T. (1965) The Effect of Amitrole upon Nucleic Acid and Protein Metabolism of Wheat Seedlings. *Physiologia Pl.*, **18**, 805.

BROCKMAN, R. W., SHADDIX, S., LASTER, W. R. JR & SCHNABEL, F. M. JR. (1970) Inhibition of Ribonucleotide Reductase, DNA Synthesis, and L1210 Leukemia by Guanazole. *Cancer Res.*, **30**, 2358.

FANG, S. C., GEORGE, M. & CHANG YU, T. (1964) Metabolism of 3-amino-1,2,4-triazole-5-$C^{14}$ by Rats. *J. agric. Fd Chem.*, **12**, 219.

FANG, S. C., KHANNA, S. & RAO, A. V. (1966) Further Study on the Metabolism of Labelled 3-amino-1,2,4-triazole (ATA) and its Plant Metabolites in Rats. *J. agric. Fd Chem.*, **14**, 262.

HAHN, M.-A. & ADAMSON, R. H. (1972) Pharmacology of 3,5-diamino-1,2,4-triazole (Guanazole). 1. Antitumor Activity of Guanazole. *J. natn. Cancer Inst.*, **48**, 783.

HEIM, W. G., APPLEMAN, D. & PYFROM, H. T. (1955) Production of Catalase Changes in Animals with 3-amino-1,2,4-triazole. *Science, N.Y.*, **123**, 693.

HILTON, J. L. (1969) Inhibitions of Growth and Metabolism by 3-amino-1,2,4-triazole (Aminotriazole). *J. agric. Fd Chem.*, **17**, 182.

HOSHIMO, M. (1960) Effect of 3-amino-1,2,4-triazole on the Experimental Production of Liver Cancer. *Nature, Lond.*, **186**, 174.

HULANICKA, D., KLOPOTOWSKI, T. & BAGDASARIAN, G. (1969) Inhibition of Aminotriazole Ribotide Biosynthesis in *Salmonella typhimurium* by Aminotriazole. *Acta biochim. Polon*, **16**, 127.

INNES, J. R. M., ULLAND, B. M., VALERIO, M. G., PETRUCELLI, L., HART, E. R., PALOTTA, A. J., BATES, R. R., FALK, H. L., GART, J. J., KLEIN, M., MITCHELL, J. & PETERS, J. (1969) Bioassay of Pesticides and Industrial Chemicals for Tumorigenicity in Mice: A Preliminary Note. *J. natn. Cancer Inst.*, **42**, 1101.

JUKES, T. H. & SHAFFER, C. B. (1960) Antithyroid Effects of Aminotriazole. *Science, N.Y.*, **132**, 296.

KLOPOTOWSKI, T. & BAGDASARIAN, G. (1966) Partial Reversal by Purine and Pyrimidine Bases of Yeast Growth Inhibition Produced by 3-amino-1,2,4-triazole. *Acta biochim. Polon.*, **13**, 153.

LACASSAGNE, A., ROSENBERG, A.-J., XUONG, N. D. & HURST, L. (1967) Actions comparées du 3-amino-1,2,4-triazole (AT) et de l'acide oxamique (AO) sur la cancérisation du foi par le p-diméthylaminoazobenzène (DAB). *C.R. Acad. Sci. Paris*, Série D, **265**, 646.

NAPALKOV, N. P. (1962) Blastomogenic Effect of 3-amino-1,2,4-triazole. *Gig. Truda prof. Zabol.*, **6**, 48.

RECHCIGLE, M. JR, HRUBAN, Z. & MORRIS, H. P. (1969) The Roles of Synthesis and Degradation in the Regulation of Catalase Levels in the Neoplastic Tissues. *Enzymol. biol. & clin.*, **10**, 161.

TJÄLVE, H. (1974) The Distribution of Aminotriazole in Mice. *Toxicology*. In the press.

ULLBERG, S. (1954) Studies on the Distribution and Fate of $S^{35}$-labelled Benzylpenicillin in the Body. *Acta radiol.* Suppl., **118**, 1.

ULLBERG, S. (1958) Autoradiographic Studies on the Distribution of Labelled Drugs in the Body. *Second U.N. Int. Conf. Peaceful Uses of Atomic Energy*, **24**, 248.

WOLF, F. T. (1962) Growth Inhibition of Chlorella Induced by 3-amino-1,2,4-triazole, and its Reversal by Purines. *Nature, Lond.*, **193**, 901.